# An NRF2 Perspective on Stem Cells and Ageing

**DOI:** 10.3389/fragi.2021.690686

**Published:** 2021-06-15

**Authors:** Matthew Dodson, Annadurai Anandhan, Donna D. Zhang, Lalitha Madhavan

**Affiliations:** ^1^ Department of Pharmacology and Toxicology, University of Arizona, Tucson, AZ, United States; ^2^ Department of Neurology, University of Arizona, Tucson, AZ, United States; ^3^ Evelyn F. McKnight Brain Institute and Bio5 Institute, University of Arizona, Tucson, AZ, United States

**Keywords:** NRF2, ageing, neural stem cells, redox, metabolism

## Abstract

Redox and metabolic mechanisms lie at the heart of stem cell survival and regenerative activity. NRF2 is a major transcriptional controller of cellular redox and metabolic homeostasis, which has also been implicated in ageing and lifespan regulation. However, NRF2’s role in stem cells and their functioning with age is only just emerging. Here, focusing mainly on neural stem cells, which are core to adult brain plasticity and function, we review recent findings that identify NRF2 as a fundamental player in stem cell biology and ageing. We also discuss NRF2-based molecular programs that may govern stem cell state and function with age, and implications of this for age-related pathologies.

## Introduction

The importance of redox and metabolic states in regulating stem cell homeostasis has become increasingly clear in recent years (Folmes et al., 2012; Bigarella et al., 2014; Holmström and Finkel, 2014; Perales-Clemente et al., 2014). Stem cells, including pluripotent and adult stem cells, have been shown to possess unique redox and metabolic programs that control their survival and function (Zhang J. et al., 2012; Ito and Suda, 2014; Zhong et al., 2019). These programs are essential to maintaining the balance between stem cell self-renewal, proliferation, and differentiation, which is fundamental to their regenerative capacity. Accordingly, it has been found that redox and metabolic genes are among the most enriched transcripts and proteins present in stem cells (Zhang J. et al., 2012; Ito and Suda, 2014; Zhong et al., 2019).

Nuclear factor (erythroid-derived 2)-like 2 (NRF2) is a common upstream regulator of redox and metabolic genes. NRF2 is a member of a family of basic leucine transcription factors that binds to Antioxidant Response Elements (AREs) in the promoter region of genes involved in redox regulation, proteostasis, DNA repair, prevention of apoptosis, iron and heme metabolism, and phase I, II, and III drug/xenobiotic metabolism (Hayes and Dinkova-Kostova, 2014; Yamamoto et al., 2018; Dodson et al., 2019; Schmidlin et al., 2019). NRF2 transcriptional responses are critical not only in maintaining normal homeostasis, but also restoring it following a range of oxidative and xenobiotic insults. Besides its classical role in modulating the stress response, NRF2 can also control basic cellular functions such as cell proliferation and differentiation, mitochondrial function, and protein quality control (Sykiotis and Bohmann, 2008; Tullet et al., 2008; Malhotra et al., 2010; Wakabayashi et al., 2010; Holmström et al., 2013; Wiesner et al., 2013; Zhu et al., 2013). Additionally, NRF2 has been implicated in ageing and longevity processes (Sykiotis and Bohmann, 2008; Tullet et al., 2008).

More broadly, it is known that deviations in redox and metabolic processes can contribute to aging and age-associated pathologies. Reactive oxygen species (ROS), including superoxide, hydrogen peroxide, hydroxyl radical, and peroxynitrite levels rise with age, and oxidative and metabolic changes are recognized contributors to several disorders of aging, including neurodegenerative disorders (Yap et al., 2009; Holmström and Finkel, 2014; Schmidlin et al., 2019). One of the key molecular cascades whose functionality decreases with age is the NRF2 signaling pathway (Zhang H. et al., 2015; Kubben et al., 2016; Schmidlin et al., 2019). However, NRF2’s importance in stem cells, their maintenance with age, and how this may relate to age-related neural pathologies, is only beginning to be revealed. In this review, with an emphasis on neural stem cells, we summarize and discuss recent findings on NRF2’s involvement in regulating stem cell fate and function during the normal lifespan and in the context of injury and age-related pathologies. This is a crucial topic to understand given the fundamental role of neural stem cells in adult neurogenesis as well as their relevance to age-related promotion of disease.

### Metabolic and Redox Regulation of Neural Stem Cells

#### Neural Stem Cell Basics

**FIGURE 1:**
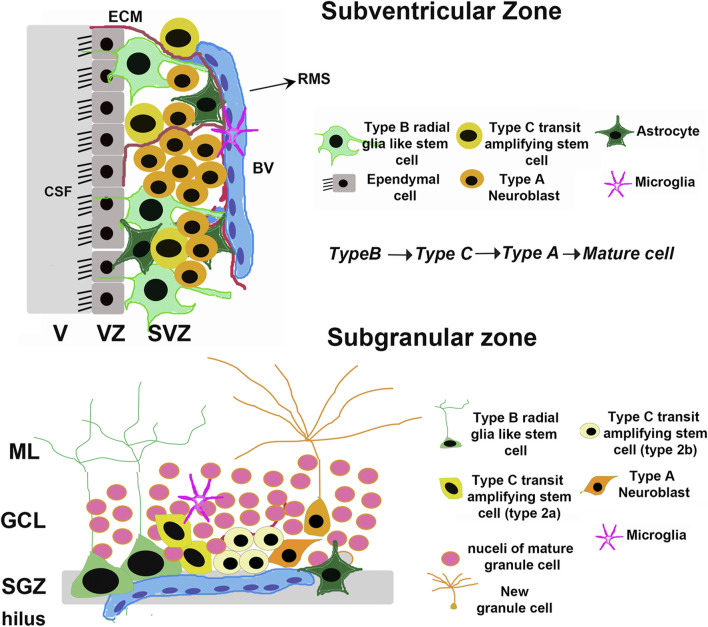
Adult neural stem cell niches. Schematic depicting the general organization of adult mammalian neural stem cell niches, taking rodents as an example. The major niches are located in the Subventricular Zone (SVZ) in the forebrain and Subgranular Zone (SGZ) in the dentate gyrus of the hippocampus. V, Ventricle; VZ, Ventricular zone; CSF, Cerebrospinal fluid; BV, Blood vessel; ECM, Extracellular Matrix; RMS, Rostral Migratory Stream; GCL, Granule Cell Layer; ML, Molecular Layer.

The majority of neural stem cells (NSCs) that can undergo adult neurogenesis in the brain are present in either the subventricular zone (SVZ) adjacent to the lateral ventricles, or the subgranular zone (SGZ) of the hippocampal dentate gyrus (DG) (Figure 1). In both the SVZ and the SGZ, the most undifferentiated stem cells (type B) are a subset of glial astrocytes in terms of their structural and electrophysiological properties (Riquelme et al., 2008). In both regions, NSCs also show a hierarchy of division: glial-like type B cells divide relatively infrequently to give rise to rapidly dividing type C transit-amplifying cells, which in turn generate immature type A neuroblasts that mature into fully differentiated neurons (Riquelme et al., 2008) (Figure 1). Type A neuroblasts in the SVZ classically migrate via the rostral migratory stream (RMS) to the OB where they mature into inhibitory neurons that fully integrate into the existing OB circuitry (Riquelme et al., 2008). On the other hand, new SGZ neurons migrate only a short distance into the granule cell layer (GCL) and integrate into the DG circuitry by extending dendrites into the molecular layer (Corenblum et al., 2016) and an axon along the mossy fiber path. The integration of immature neurons in both the OB and hippocampus have behavioral significance (Sahay et al., 2011b). Olfactory neurogenesis is important for olfactory perception, whereas hippocampal neurogenesis is required for certain cognitive tasks, including learning, memory processing, pattern separation, and mood regulation (Gould et al., 1999; Higashi et al., 2008; Sahay et al., 2011a; Sahay et al., 2011b). As such, the neurogenic capacity of the adult brain depends in large part on the ability of these two NSC populations to renew and differentiate into functional mature cell types when needed. The bulk of NSCs in both the SVZ and SGZ are present in a quiescent state, undergoing self-renewal via a very slow cell cycle designed to maintain the population throughout post-embryonic life; however, upon the receipt of certain stimuli, these quiescent NSCs can become activated and begin proliferating at a much more rapid rate. This population of more rapidly proliferating NSCs, termed neural progenitor cells (NPCs), can in turn differentiate into neurons, astrocytes, or oligodendrocytes depending on the cue received (Bond et al., 2015).

#### Metabolic Regulation

Much like other adult stem cell populations (i.e. mesenchymal stem cells, hematopoietic stem cells, etc.), as well as the differentiated cell types they become, proper metabolite utilization via primary metabolic pathways is a key regulator of NSC survival and regenerative function. For example, while quiescent NSCs are thought to mainly utilize glycolysis or fatty acid oxidation to meet the low metabolic requirements of dormancy, activation and differentiation towards a neuronal lineage is associated with more reliance on oxidative phosphorylation (Stoll et al., 2015; Zheng et al., 2016; Knobloch et al., 2017, Figure 2). In fact, single cell RNA sequencing of transcriptomic differences between quiescent and activated NSCs revealed that gene expression of glycolytic and fatty acid oxidation enzymes is increased during quiescence, switching over to a more oxidative phosphorylation-based transcriptional program upon activation (Shin et al., 2015). Interestingly, increased reliance on mitochondrial metabolism to exit the quiescent state is also associated with significant alterations to the morphology, mass, distribution, and overall membrane potential of the mitochondrial network (Beckervordersandforth et al., 2017). This indicates that NSC activation and subsequent differentiation results in an increased reliance on mitochondrial metabolism, presumably to meet the additional energetic requirements of a non-quiescent state.

**FIGURE 2:**
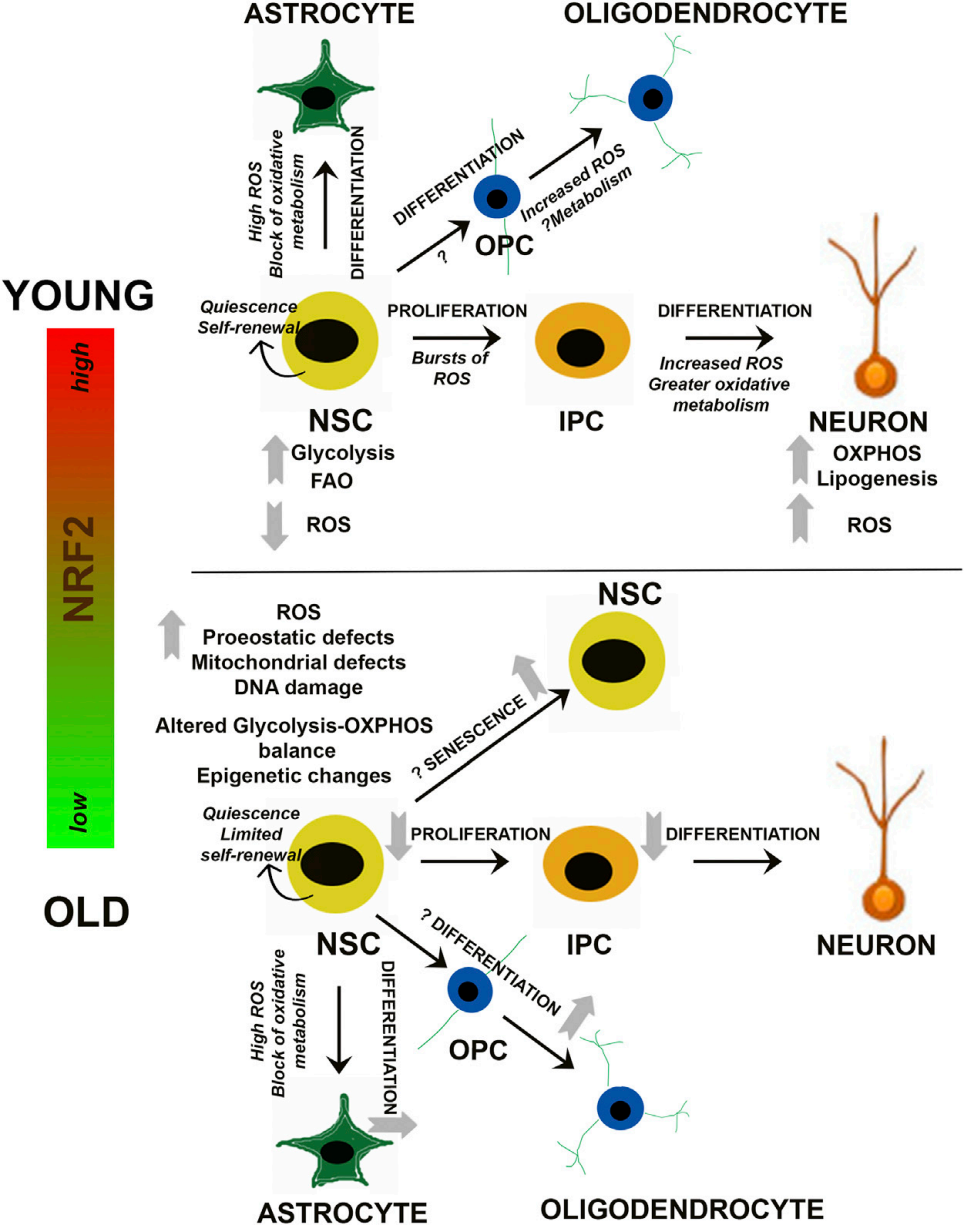
Redox and Metabolic Regulation of NSCs. Schematic summarizing current findings on how changes in NRF2-based ROS and metabolic processes may influence the balance between NSC quiescence, self-renewal and differentiation. Alterations during NSC differentiation into neuronal and glial (astrocytes and oligodendrocytes) cells is depicted, and the questions marks indicate aspects which are yet unknown. IPC, Intermediate progenitor cell; OPC, Oligodendrocyte progenitor cell.

Along with glycolysis and mitochondrial bioenergetics, lipogenesis and fatty acid mobilization from lipid droplets have also been shown to play a key role in NSC/NPC fate determination. Fatty acid synthase (*Fasn*), which is a key regulator of *de novo* lipogenesis, is highly expressed in NPCs, and conditional knockout of *Fasn* in mouse NPCs has been shown to prevent neurogenesis (Knobloch et al., 2013). Similarly, inherent deficiencies in the ability to mobilize fatty acids, particularly from lipid droplets, has been shown to decrease the number of NPCs in the embryonic mouse neocortex (Xie et al., 2016). At the epigenetic level, control of cell fate has been linked to the NAD^+^: NADH ratio, with the sirtuin family of deacetylases playing a central role. Specifically, sirtuin 1 (SIRT1) levels closely correlate with NSC activation, as SIRT1 levels are increased in activated NSCs. Contrastingly, the proliferation and self-renewal rate of hippocampal NSCs were elevated in *Sirt1* ^−/−^ mice (Hisahara et al., 2008; Ma et al., 2014). Thus, it appears that the switch from a quiescent to more active/differentiated state in NSCs involves downregulation of glycolysis and fatty acid oxidation, followed by a switch to oxidative phosphorylation and increased lipogenesis. This shift is coupled to significant changes in mitochondrial morphology, reactive oxygen species (ROS) production, and metabolically driven epigenetic changes that ultimately determine NSC/NPC fate (Figure 2).

#### Redox Regulation

It is known that oxidation-reduction mechanisms are vital regulators of cellular survival and function. In stem cells, studies support an important role for redox sensitive signaling networks, which control its signature properties of self-renewal, proliferation, and differentiation in multiple organ systems such as bone, blood, and the brain (Bigarella et al., 2014; Holmström and Finkel, 2014). With respect to the nervous system, cellular redox status is recognized as a central regulator of NSC function (Smith et al., 2000; Noble et al., 2003; Hochmuth et al., 2011; Rafalski and Brunet, 2011). Even minute alterations in the redox state can radically affect stem/progenitor function. More specifically, in oligodendroglial progenitors, it has been reported that a highly reduced intracellular redox state promotes proliferation and survival, whereas a highly oxidized state results in more differentiation and apoptosis. In addition, NSC proliferation can be enhanced by factors like peroxiredoxins, glutathione peroxidase 1 (GPX1), and sestrin 3, which can control reactive oxygen species (ROS) and thus redox state (Paik et al., 2009). In addition, the energy-sensing deacetylase SIRT1, mentioned above, has been reported to be activated under oxidative conditions and alter the differentiation of embryonic mouse NSCs so that astrocytes rather than neurons are generated (Prozorovski et al., 2008; Madhavan, 2015b) (Figure 2).

As one might expect, the shift to a more oxidative state during differentiation comes with an increased risk of generating more reactive species. However, while increased ROS is known to be detrimental to cell survival and function across a variety of contexts, NSC oxidant production plays an integral role in mediating the balance between self-renewal and differentiation. For example, it has been reported that more proliferative NSCs exhibit higher basal ROS levels than their less proliferative, quiescent counterparts, and that endogenous production of ROS (i.e. via NADPH oxidase 2 (NOX2)-dependent production of superoxide and hydrogen peroxide) is also necessary for PI3K/AKT-dependent NSC self-renewal (Le Belle et al., 2011). Intriguingly, NOX2-dependent production of superoxide is also essential for NSC differentiation into a more neuronal phenotype in the brain of adult newts, and plays an important role in promoting neurogenesis during ischemic injury (Hameed et al., 2015). Supporting the notion that ROS is necessary for NSC maintenance in a mammalian setting, treatment of embryonic mouse NSCs with a variety of antioxidants was shown to decrease their proliferative capacity, whereas the peroxynitrite generator SIN-1 enhanced proliferation (Yoneyama et al., 2010). Despite the clear role that a pro-oxidative shift in metabolism has in driving NSC proliferation/differentiation, it appears there is also a limit to how much ROS can be tolerated, as a number of studies have indicated that loss of superoxide dismutase (SOD) 1, 2, or 3 can bias NSC differentiation towards more astrocyte formation, decrease hippocampal neurogenesis, and impair cognitive function in irradiated mice (Rola et al., 2007; Fishman et al., 2009; Raber et al., 2011). Even mitochondrial dynamics, which can be dictated by the redox environment, have been shown to play an integral role in dictating ROS-driven NSC cell fate decisions. Specifically, self-renewing NSCs have fused mitochondria and low basal ROS levels; however, upon differentiation mitochondria become more fragmented and ROS levels are increased (Khacho et al., 2016).

Overall, these studies support the notion that achieving a “certain” appropriate redox balance is an important determinant of NSC state and function. These results also highlight the complexity of the redox response, which may be quite diverse depending on the precise context. Such an intricate regulation by oxygen would allow NSCs to not only adapt to environmental changes occurring under normal physiological conditions during their lifespan, but also enable them to be acutely sensitive to pathological stressors posed during disease and quickly respond by increasing their regenerative capacity and accelerating tissue repair (Madhavan, 2015a; Madhavan, 2015b). In this regard, some of our own work has compared NSCs and mature neural cell types in terms of their sensitivity to oxidative stress (Madhavan et al., 2006). More specifically NSCs, from the early postnatal brain, and their differentiated neuronal and glial counterparts, were exposed to 3-nitropropionic acid (3-NP) which induces the generation of harmful ROS such as hydroxyl radicals, resulting in increased oxidative stress. It was found that undifferentiated NSCs, at a basal level, harbored lower ROS and higher antioxidants as compared to mature neural cells. Additionally, when the NSCs were challenged with 3-NP, NSCs quickly increased their expression of antioxidants such as glutathione peroxidase (GPx), superoxide dismutase 2 (SOD2), and uncoupling protein 2 (UCP2) compared to their differentiated counterparts. Furthermore, when the postnatal NSCs were implanted into the striatum of mice treated with 3-NP, they not only maintained high intrinsic SOD2 levels themselves, but were also able to express specific trophic factors such as ciliary neurotrophic factor (CNTF) and vascular endothelial growth factor (VEGF) to activate SOD2 in the endangered host striatal neurons (Madhavan et al., 2005; Madhavan et al., 2006; Madhavan et al., 2008). This ultimately led to neuroprotection and improved striatal function in the 3-NP treated animals.

These findings indicate that an acute sensitivity to redox alterations may be a key intrinsic feature of NSCs which allows them to adapt to changes in their environment and maintain their regenerative function under physiological and pathological circumstances. As such, it is not a surprise that many of the therapies proposed to target NSC loss of function and depletion may provide their beneficial effects through enhancing mitochondrial functionality to prevent excessive oxidative stress, including NAD^+^ supplementation, metformin-induced mitochondrial biogenesis, and antioxidant supplementation (Khacho et al., 2019).

#### Comparisons to Other Stem Cells

Much like NSCs, other adult stem cell types have also been shown to rely on redox and metabolic changes to maintain self-renewal or trigger differentiation. For example, similar to dormant NSCs, hematopoietic stem cells (HSCs) have low mitochondrial activity and mainly rely on HIF-1α-driven glycolysis, which is attributed to residing in a hypoxic niche and the low metabolic needs of quiescence (Simsek et al., 2010; Takubo et al., 2013). As was reported with NSCs, fatty acid oxidation is also important in HSC maintenance, as inhibition of fatty acid oxidation resulted in a loss of HSC self-renewal (Ito et al., 2012). Interestingly, low oxygen levels are also required to maintain embryonic stem cells (ESCs) and muscle stem cells (MuSCs) in a self-renewing, undifferentiated state (Mazumdar et al., 2010; Majmundar et al., 2012). Consistent with this, ROS levels have been linked to the differentiation status of a number of stem cell types including ESCs and HSCs, with lower ROS driving self-renewal, and higher ROS levels being associated with differentiation (Jang and Sharkis, 2007; Bigarella et al., 2014). Similarly, mesenchymal stem cells (MSCs) have also been shown to exhibit high antioxidant capacity and low ROS levels; however, their differentiation into a variety of mesenchymal cell types requires increased production of superoxide and hydrogen peroxide by mitochondrial complexes I and III, as well as NADPH oxidase 4 (NOX4) (Atashi et al., 2015). Thus, along with NSCs, the balance between self-renewal and differentiation in other stem cell types is also clearly regulated by metabolic switches that govern ROS production.

### NRF2 as a Regulator of Stem Cells

#### In Physiological Conditions

As mentioned above, NRF2 is a critical transcription factor that has been shown to regulate target genes involved in almost every facet of metabolism, ranging from maintaining the redox balance to ensuring proper protein quality control (Dodson et al., 2019). Accordingly, NRF2 has been shown to drive important aspects of embryonic, adult, and induced-pluripotent stem cell proliferation, differentiation, and function. Jang et al., have reported that human embryonic stem cells (hESCs) express high levels of NRF2, and that self-renewal and eventual cellular reprogramming of hESCs relies on increased proteasomal activity, which is driven in part by NRF2-dependent upregulation of proteasome maturation protein (*POMP*) (Jang et al., 2014). Later, findings from the same group demonstrated that differentiation of hESCs towards a neuroectoderm fate requires primary cilia-autophagy-dependent degradation of NRF2, which results in decreased expression of *OCT4* and *NANOG* and increased differentiation towards a neuronal fate (Jang et al., 2016). Similar to hESC differentiation, metabolic reprogramming of induced pluripotent stem cells (iPSCs) has also been shown to rely on NRF2. Specifically, iPSC reprogramming is driven by an initial oxidative burst that results in the ROS-dependent activation of NRF2. NRF2 then upregulates its downstream target HIF-1α to shift glucose utilization away from glycolysis towards the pentose phosphate pathway (Hawkins et al., 2016). Along these lines, the same study showed that KEAP1 overexpression, which suppresses NRF2 levels, also decreased iPSC colony formation and cellular reprogramming. Furthermore, NRF2 mRNA levels and transcriptional activity were also shown to steadily increase throughout the iPSC differentiation process, peaking during the later stages (Zhou et al., 2016). Overall, these studies indicate a key role for NRF2 in dictating the ability of hESCs and iPSCs to shift from a pluripotent to a more differentiated state.

Much like their more pluripotent counterparts, a number of adult stem cell (Halaschek-Wiener and Brooks-Wilson, 2007) types have also been shown to rely on the NRF2 pathway to maintain their ability to self-renew and differentiate. For instance, the ability of mesenchymal stem cells (MSCs) to undergo osteogenesis is inhibited by loss of NRF2, whereas overexpression increases MSC proliferation (Yuan et al., 2017). NRF2 regulation of the osteogenic potential and self-renewal capability of MSCs could rely upon its activation of SIRT1, which is controlled at the transcriptional level by NRF2-mediated suppression of p53. On the other hand, NRF2-dependent activation of SIRT1 increases MSC self-renewal and osteogenic potential, whereas knockdown of NRF2 has the opposite effect (Yoon et al., 2016). The governance of stem cell function by an NRF2-sirtuin axis is further supported by evidence that SIRT6 knockout MSCs exhibited a functional decay driven by redox abnormalities and increased sensitivity to oxidative stress (Pan et al., 2016). This redox imbalance was a direct result of SIRT6 being required for NRF2 transcriptional activation of a certain subset of target genes, including heme-oxygenase 1 (HO-1), and overexpression of HO-1 was able to rescue MSC attrition. A number of other adult stem cell types, including hematopoietic stem cells (HSCs), airway basal stem cells (ABSCs), and glioma stem cells (GSCs), have all been shown to rely on NRF2 activation to properly self-renew (Tsai et al., 2013; Zhu et al., 2013; Paul et al., 2014). Conversely KEAP1-dependent repression of NRF2 is required for intestinal stem cells (ISCs) from *Drosophila* to proliferate, although complete loss of NRF2 results in ROS accumulation and intestinal degeneration (Hochmuth et al., 2011). Long-noncoding RNA ROR has also been shown to suppress NRF2 levels in mammary epithelial cells (MaSCs), which leads to increased self-renewal and maintenance of MaSCs *in vivo* (Zhang et al., 2014). Thus, while NRF2 clearly participates in determining the balance between ASC self-renewal and activation/differentiation, the exact role it plays is context and stem cell niche dependent.

#### In Neurological Injury and Pathology

In addition to NRF2’s regulation of NSC physiology and function discussed above, a number of studies have revealed an important role for NRF2 in dictating NSC viability and function during stress-induced neurogenesis. *Nrf2* ^*−/−*^ mice have been shown to have impaired hippocampal neurogenesis following ischemic injury, with overexpression or pharmacological activation of NRF2 using dithiocarbamate enhancing NSC proliferation and differentiation *in vitro* (Kärkkäinen et al., 2014). Also, the transplantation of rat NSCs preconditioned with the antibiotic minocycline or doxycycline can also protect against ischemic injury *in vivo* via upregulated NRF2 (Sakata et al., 2012; Malik et al., 2013). Building on this notion that NRF2 induction is necessary for neural stem/progenitor cell survival and differentiation upon oxidative insult, pretreatment with the canonical NRF2 inducers tert-butylhydroquinone (tBHQ) or dimethyl fumarate (DMF) significantly attenuated H_2_O_2_-induced cell death in rat NPCs (Li et al., 2005; Wang et al., 2015). With regards to oxidant-driven differentiation of NSCs, exposure to the naphthoquinone derivate 1a resulted in an NRF2/SIRT1-dependent shift away from a neurogenic lineage towards a gliogenic fate, which was prevented by the addition of exogenous antioxidants (Santos et al., 2013). Similarly, differentiation of human iPSC-derived NSCs into neurons and glia has been shown to correlate with increased NRF2 signaling, presumably to compensate for the shift to a more oxidative metabolism, as mentioned above. However, repeated exposure to the mitochondrial superoxide generator rotenone during NSC differentiation results in the decreased production of neuronal cell types, including dopaminergic neurons (Pistollato et al., 2017), inferring that NRF2 activation may not be able to fully compensate for excessive exogenous insult. Either way, these studies clearly indicate that NRF2 is critical in maintaining the neurogenic capacity of NSCs, particularly in the presence of increased oxidative stress. Furthermore, loss of neurogenesis, whether it be a result of age or injury, mainly results from decreased survival or proliferative capacity of the different NSC populations, with activation of the NRF2 pathway clearly playing a protective role.

### NRF2 Role in Longevity, Ageing, and Age-Related Stem Cell Function

#### In Longevity

NRF2’s classic role in responding to cytotoxic stressors is well defined. Nevertheless, only recently have studies begun to address NRF2 function in other contexts such as aging and lifespan regulation. For example, involvement of the NRF2 homolog SKN-1 in *Caenorhabditis elegans (C. elegans)*, and the *Drosophila* homolog CncC, in determining longevity was recently shown. Upon activation, SKN-1 and CncC upregulate genes involved in the oxidative stress response, including many orthologs of those regulated by mammalian NRF2 (An et al., 2005; Sykiotis and Bohmann, 2008), and can significantly increase longevity (Sykiotis and Bohmann, 2008; Tullet et al., 2017). Also, loss of SKN-1 shortens lifespan, whereas SKN-1 gain-of-function usually extends lifespan (Blackwell et al., 2015; Tang et al., 2015). Additionally, in the *daf*-2 (nematode homologue of FOXO) mutant, which is long lived, increases in lifespan occur via the activation of SKN-1 (Tullet et al., 2008). Similarly, the lifespan of male *Drosophila* can be extended by KEAP-1 loss-of-function mutations (Sykiotis and Bohmann, 2008). Interestingly, in birds (which also have long lifespans), a constitutive activation of the NRF2 antioxidant response has been reported. Such NRF2 activation provides an adaptive mechanism capable of controlling naturally elevated ROS levels in these species due to high metabolic demands (Castiglione et al., 2020). It has been found that this adaptation involves an ancient KEAP 1 mutation that initiates a compensatory program in NRF2 target genes to control redox status as well as other fundamental aspects such as feather development. With regards to rodents, cells obtained from the extremely long-lived naked mole rat, have been shown to have considerably high NRF2 and downstream target gene activity, proteasome activity, and oxidative stress resistance (Blackwell et al., 2015). It has also been shown that deletion of glutathione transferase (*Gsta4*) can activate NRF2 and significantly increase mouse lifespan (Singh et al., 2010). Additionally, slowing of aging via genetic, dietary, or pharmacological interventions results in the upregulation of NRF2 targets and other detoxification genes in multiple rodent models (Swindell, 2007; Leiser and Miller, 2010). Intriguingly, fly studies show that while mild NRF2 activation extends lifespan, sustained NRF2 activation can be lethal and drastically reduce longevity (Tsakiri et al., 2019). This contradiction suggests that the activation level of NRF2 that enhances health and lifespan may be considerably lower than that which promotes cytoprotection. Nevertheless, all in all, these studies suggest that mechanisms regulated by NRF2 may be of conserved importance in longevity processes.

#### In Ageing

Despite its ability to promote longevity, NRF2 is ironically suppressed during aging—a time when the organisms might most need its function. Evidence for this phenomenon was initially reported in rat studies showing that basal NRF2 levels decline with age (Sykiotis and Bohmann, 2010). Such NRF2 reduction correlates with decreased expression of its target genes, lower intracellular glutathione, and increased generation of oxidation products (Suh et al., 2004; Sykiotis and Bohmann, 2010). In fact, aged mice appear to show similar losses in cellular redox capacity to those observed in NRF2 knockout mice (Suh et al., 2004; Kensler et al., 2007). Disruptions in NRF2 signaling have also been reported in sedentary older humans (Safdar et al., 2010). Specifically, in skeletal muscle, age-associated reductions in NRF2 target gene expression is accompanied by increased hydrogen peroxide and 4-hydroxynonenal (4-HNE) production, glutathione depletion, and oxidant damage (Safdar et al., 2010). These data indicate that the failure to dynamically adjust NRF2 expression and transcriptional activity may contribute to the loss of homeostasis and affect function.

Multiple mechanisms including loss-of-function mutations in the genes encoding NRF2 or small Mafs, oxidation or epigenetic modification of the NRF2 promoter, or changes in the other factors that modulate NRF2 (such as kinases, phosphatases, or DNA binding proteins) have been suggested to contribute to the suppression of NRF2 activity during ageing. Interestingly, in naked mole rats, it was found that longevity was not linked to the protein levels of NRF2 itself, but rather showed a notable negative relationship with the regulators KEAP1 and β-transducin repeat-containing protein (βTrCP), which target NRF2 for degradation (Lewis et al., 2015). Regardless of the precise mechanism, it can be conceived that organismal aging may promote specific pathologies through the suppression of the stress response and loss of homeostasis in relevant organs. Conversely, the impairment of NRF2 activity during disease may accelerate the age-related deterioration of the affected tissue (Sykiotis and Bohmann, 2010).

#### In Stem Cell Ageing

An important part of the aging phenotype related to NRF2 loss involves stem cells. In this regard, our own studies have identified that the age-dependent decline in SVZ NSC function and survival is associated with a loss of NRF2, particularly during a critical middle age period spanning from ∼13 to 15 months of age (Corenblum et al., 2016; Ray et al., 2018; Anandhan et al., 2021). During this critical time, NSC proliferation and neuronal differentiation significantly declines, but their potential to generate astrocytes increases. Interestingly, we also found that siRNA-mediated knockdown of NRF2 in “young” SVZ NPCs promoted a shift toward the decreased survival and regenerative characteristics of “old” NPCs, with overexpression of NRF2 in old NPCs promoting a reversion to a younger phenotype (Corenblum et al., 2016). This reliance of NSCs on proper NRF2 function was further confirmed using an *Nrf2* ^*−/−*^ mouse, which exhibited fewer SVZ NPCs than wild-type mice, with surviving *Nrf2* ^*−/−*^ NSCs proliferating less and exhibiting decreased neurogenic potential (Corenblum et al., 2016; Ray et al., 2018).

On the other hand, NRF2 expression does not seem to affect the viability of SGZ NPCs as they age; however, the ability of this population of NPCs to proliferate and differentiate, particularly during the critical middle age period, is reliant on NRF2 similar to their SVZ counterparts. In fact, age-related loss of hippocampal function can be restored by transplantation of NRF2-overexpressing young but not old NSCs, indicating that NRF2 is a critical mediator of NSC/NPC-dependent neurogenesis with age (Ray et al., 2018). Furthermore, our recent work indicates that the viral overexpression of NRF2 in the rodent SVZ can mitigate the decline in NSC function across the critical middle-age period (Anandhan et al., 2021). Along these lines, a similar study from Antonio Cuadrado’s group showed that NSCs isolated from the SGZ of *Nrf2* ^*−/−*^ mice have decreased clonogenic and neurogenic potential, as well as an impaired capacity for long-term progression from birth to adulthood in *Nrf2* ^*−/−*^ mice compared to wild-type controls (Robledinos-Antón et al., 2017).

NRF2 contributions to the ageing of other types of stem cells has also been reported. For instance, a key mechanism in the premature aging disease, Hutchinson-Gilford progeria (HGPS), seems to involve NRF2. It was shown that NRF2 is ensnared by progerin resulting in its impaired signaling, chronic oxidative stress, and an aging phenotype (Kubben et al., 2016). Restoring NRF2 activity in iPSC-derived MSCs from HGPS patient fibroblasts increased their viability, indicating that NRF2 activation maybe useful in counteracting the premature exhaustion of adult stem pools normally seen in HGPS patients (Halaschek-Wiener and Brooks-Wilson, 2007; Liu et al., 2011; Zhang et al., 2011). On the other hand, in *Drosophila* ISCs, loss of NRF2 causes ROS accumulation and accelerates the age-related degeneration of the intestinal epithelium (Hochmuth et al., 2011). The underlying cause appears to be a compromise in ISC capacity to engage an important proteostatic checkpoint, a mechanism that can be restored by treating flies with an NRF2 activator, or by over-expression of CncC or Atg8a. This NRF2-based intervention reduced the observed age-related intestinal barrier dysfunction and promoted lifespan extension (Rodriguez-Fernandez et al., 2019).

The dynamical balance between quiescence, proliferation, and senescence is naturally critical for sustaining organismal stem cell pools. In this context it has been found that long term hematopoietic stem cells (LT-HSCs), a rare quiescent population in bone marrow, are modulated by NRF2 signaling (Murakami et al., 2017). At steady state, a KEAP-1 deficiency based persistent activation of NRF2 reduced HSC quiescence without affecting their numbers. Persistent activation of NRF2 during hematopoietic regeneration after bone marrow (BM) transplantation, compromised the BM reconstitution capacity of LT-HSCs. These data suggest that NRF2 reduced the quiescence of LT-HSCs while promoting their differentiation, leading to eventual exhaustion. A transient activation of NRF2 was also found to promote cell cycle entry of the LT-HSCs. This indicated that under stressful conditions such as blood loss, NRF2 promotes cell cycle entry and differentiation of HSCs while compromising their quiescence and maintenance. Thus, the appropriate control of NRF2 activity by KEAP1 is vital for maintaining HSCs under physiological conditions as well as their replenishment upon stress.

The maintenance of old stem cells in a reversibly quiescent state is intricately connected to the expression or suppression of senescence. Cellular senescence is a process closely associated with age (Schultz and Sinclair, 2016; Nicaise et al., 2019). In this aspect, it has been shown that aging promotes cellular senescence in cerebral blood vessels, which is exacerbated by impaired NRF2 signaling (Fulop et al., 2018). Senescence can also be prevented in MSCs through NRF2 activation (Fang et al., 2018). Both cellular senescence and quiescence involve hallmark changes in mitochondrial and autophagic function. Given that NRF2 is known to modulate both mitochondria and autophagy (Komatsu et al., 2010; Dinkova-Kostova and Abramov, 2015; Jiang et al., 2015; Holmström et al., 2016), it will be interesting to know whether and how NRF2 may modulate these key processes in stem cells to ultimately determine their behavior and fate.

In essence, there is compelling evidence for NRF2-based redox and metabolic signaling at the core of aging and longevity pathways. Importantly, the loss of NRF2 seems central to age-associated declines in stress resistance - and even perhaps in the aging process itself.

### Implications for NRF2 Role in Age-Related Disorders

As discussed above, the neurogenic capacity of NSCs, as well as NRF2 activity as a whole, have both been shown to decrease significantly with age. Aging, along with environmental exposures and genetic mutations, continues to represent one of the biggest risk factors for a host of diseases, including neurodegenerative and other neurological disorders. An ever-increasing number of studies have identified a broad scope of underlying causes for the age-related onset of neurodegenerative disorders, including mitochondrial dysfunction, increased oxidative damage, protein aggregation, and metabolic dysfunction (Hou et al., 2019). These same pathological phenotypes have also been shown to contribute to NSC decline during aging, with activation of NRF2 signaling restoring NSC function and survival. For example, the ability to pharmacologically activate NRF2 was shown to be suppressed in iPSC-derived NSCs from Huntington’s disease patients as compared to normal subject controls, although NRF2 induction did inhibit inflammatory cytokine production in primary mouse microglia and astrocytes from YAC128 HD mice, as well as monocytes from HD patients (Quinti et al., 2017). Similarly, overexpression of NRF2 prevents the toxicity and decreased proliferation observed in Aβ-treated NPCs, with NRF2 deficiency enhancing Aβ suppression of differentiation, suggesting NRF2 induction could preserve NSC function in Alzheimer’s brains (Kärkkäinen et al., 2014). In the context of Parkinson’s disease, a study by L’Episcopo et al. (2013) revealed that there is an imbalance of the NRF2 target gene, HO-1, in the SVZ niche, starting by middle age. This imbalance is exacerbated upon treatment with the neurotoxin, 1-methyl-4-phenyl-1,2,3,6-tetrahydropyridine (MPTP), leading to a highly proinflammatory SVZ microenvironment and the dysregulation of a key pathway regulating adult NSCs, namely Wingless-type MMTV integration site (Wnt)/β-catenin signaling. A disturbance of PI3K (phosphatidylinositol3-kinase)/Akt and the Wnt/Fzd/β-catenin signaling cascades, which switch glycogen synthase kinase 3β (GSK-3β) activation on and off, were causally related to the impairment of SVZ NSCs. Furthermore, a novel non-steroidal anti-inflammatory drug, HCT1026, was able to normalize NRF2 signaling and rescue the SVZ NSC impairment. Additionally, while not age-related, Friedrich’s ataxia has also been shown to involve NRF2 regulation of NSC function, as NSC proliferation, self-renewal, and differentiation were all impaired in cortical embryonic NSCs isolated from frataxin-deficient frataxin knock-in/knockout mice (La Rosa et al., 2019). Thus, while more *in vitro* and *in vivo* evidence of a role for NRF2 in preserving the NSC pool in neurodegenerative disease is needed, these studies clearly indicate that loss of NRF2 is detrimental during age-related pathogenesis, and that NRF2 activators could be of therapeutic benefit for this subset of diseases, partly by promoting NSC survival.

### NRF2-Based Molecular Programs Governing Stem Cell Function and Ageing

**FIGURE 3:**
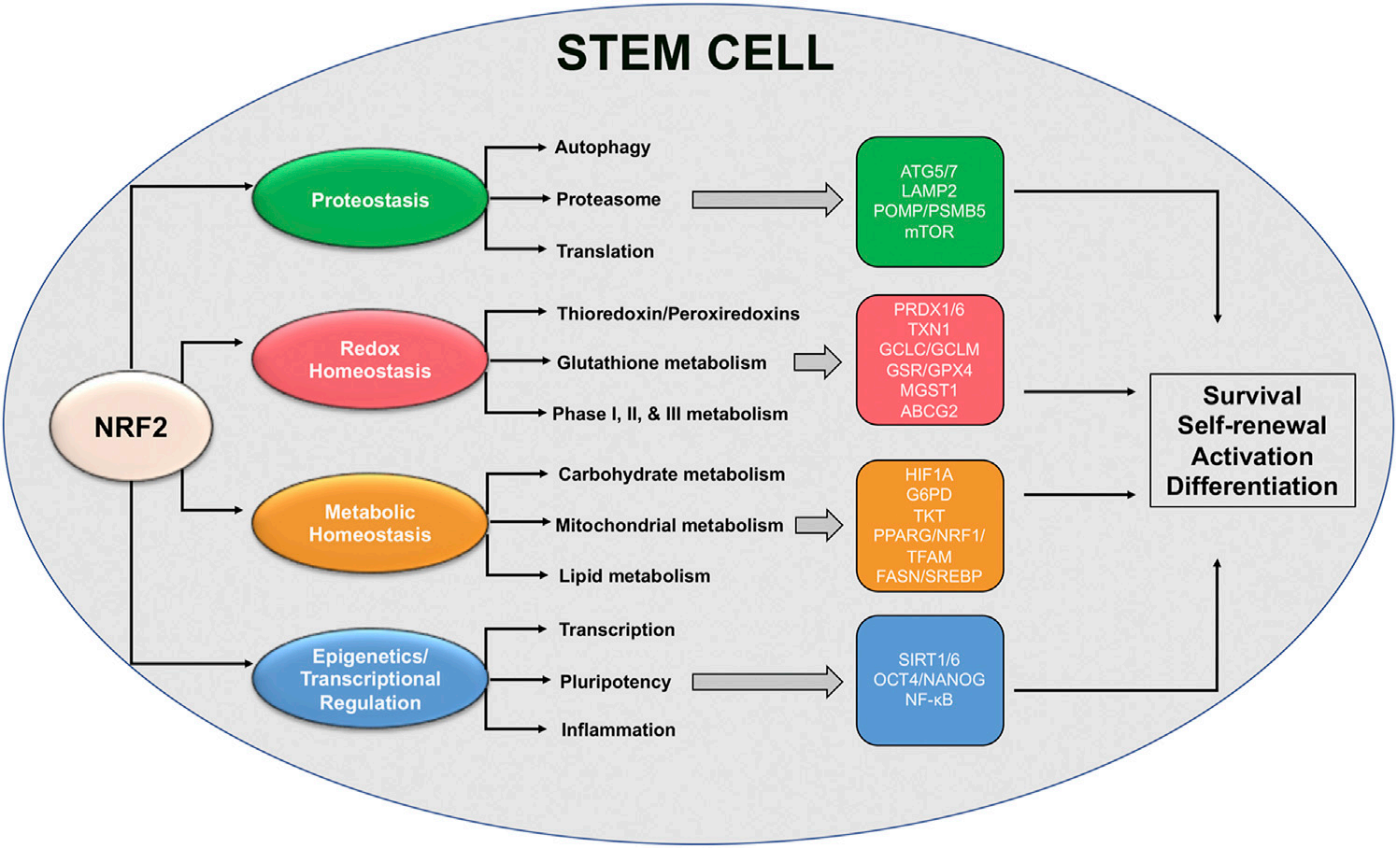
Proposed NRF2-based redox and metabolic pathways governing stem cell function. Schematic representation of NRF2-regulated pathways and specific downstream transcriptional targets that have been shown to play a role in mediating stem cell survival, self-renewal, and activation/differentiation.

As discussed earlier, NRF2 plays an integral role in mediating numerous aspects of stem cell self-renewal, activation, and differentiation. However, while a great deal of literature exists demonstrating upstream loss or activation of NRF2 can have significant consequences, very few studies have linked NRF2 with the actual downstream targets and signaling cascades that dictate its regulation of stem cell survival. This is further confounded by the fact that NRF2 target genes regulate almost all aspects of maintaining protein, redox, and metabolic homeostasis, as well as epigenetic regulators and other transcription factors; however, despite this fact, some specific target genes important for stem cell maintenance have been identified (Figure 3). In this section, we will highlight some of these downstream signaling responses, including their importance in maintaining stem cell function and the possible role of established NRF2 target genes in mediating these cascades.

#### NRF2 Regulation of Stem Cell Proteostasis

Proper protein translation, folding, and eventual degradation are essential for cell survival, particularly during stress (Higuchi-Sanabria et al., 2018). In the specific case of stem cells, alterations to protein synthesis or the various mechanisms responsible for protein degradation have been shown to have significant effects on the ability of stem cells to proliferate and differentiate when needed. In the brain, quiescent NSCs have been shown to rely mainly on the autophagy-lysosome pathway, switching over to protein maintenance via chaperones and proteasomal degradation once activated/differentiated (Leeman et al., 2018; Vonk et al., 2020). However, it is well-established that both autophagy and proteasomal function decrease with age (Saez and Vilchez, 2014), and autophagy dysfunction in particular has been linked to changes in NSC and HSC ability to exit quiescence and self-renew, respectively (Ho et al., 2017; Leeman et al., 2018). This infers that an age-dependent deterioration in neurogenic capacity could be partly driven by autophagy dysfunction-driven suppression of NSC activation. Along these same lines, loss of Atg7, an enzyme critical for autophagy initiation that is also a putative NRF2 target gene (Pajares et al., 2016), was shown to decrease the ability of HSCs to self-renew, forcing differentiation to a more myeloid lineage (Mortensen et al., 2011). Atg5, another NRF2-driven regulator of autophagy initiation, has also been shown to be increased during NSC neurogenesis in the olfactory bulb, with loss of Atg5 reducing differentiation into a neuronal phenotype (Vázquez et al., 2012). Similarly, chaperone mediated autophagy (CMA) is also increased upon HSC activation, and an age-dependent decline in CMA has been shown to correlate with HSC depletion (Dong et al., 2021). Importantly, LAMP2, which plays a critical role in mediating lysosomal degradation of CMA substrates, was also reported to be transcriptionally regulated by NRF2 (Pajares et al., 2018). Thus, NRF2 target genes appear to play a critical role in mediating the autophagy-dependent effects that regulate stem cell survival.

Similar to decreased autophagic capacity over time, proteasomal function has also been shown to decline with age. This was demonstrated in aged NSCs, as older NSCs exhibit much lower mRNA levels of *PSMB5*, an NRF2 target gene that encodes the 20S subunit of the 26S proteasome, reducing proteasome activity (Kwak et al., 2003; Zhao et al., 2016). Discussed earlier, NRF2-dependent upregulation of *POMP* is needed for hESC self-renewal and reprogramming and its expression decreases as hESCs become more differentiated (Jang et al., 2014). Intriguingly, even changes in protein translation in stem cells may be driven, at least in part, by NRF2, as mTOR a known regulator of protein synthesis also has a functional ARE (Bendavit et al., 2016). mTOR has been shown to play an important role in mediating self-renewal, cell fate determination, and differentiation of NSCs, MSCs, ISCs, hESCs, MaSCs, and HSCs (Meng et al., 2018). Thus, NRF2 levels could be a key determinant of mTOR-dependent protein translation across a variety of stem cell contexts, including NSCs and aging. However, it is important to note that the NRF2-mTOR signaling axis has not been fully validated, and more studies confirming the interaction between these two critical cell survival pathways are still needed.

#### NRF2 Controlled Redox Enzymes in Mediating Stem Cell Maintenance

As NRF2 was originally identified as a master regulator of cellular antioxidant defense, it is not surprising that many of its identified target genes are involved in preventing and mitigating the harmful effects associated with increased oxidative stress. Critically, ROS production, at least to a certain extent, is needed for many stem cell types to progress from quiescence to activation and differentiation. Thus, NRF2 regulation of redox based target genes must be fine-tuned to meet the functional demands of the stem cell given its dormant or more active state. Intriguingly, very few studies have elucidated the pathways downstream of NRF2 that are responsible for NSC maintenance and survival; however, this has been done in other stem cells contexts, which could presumably overlap with critical NSC pathways. One example of NRF2 control of the redox balance in stem cells can be seen with peroxiredoxins 1 and 6 (PRDX1/6), which are critical reducers of intracellular hydrogen peroxide. In hESCs, stemness has actually been shown to depend on suppression of ROS, as deletion of PRDX1 in hESCs results in ROS overload and decreased stemness (Kim et al., 2014). Contrastingly, a decrease in the levels of PRDX6 is required for dental pulp stem cells (DPSCs) to be able undergo osteogenic differentiation, as overexpression of PRDX6 repressed dental bone development (Park et al., 2019). This disparity once again illustrates that different stem cell niches require differing levels of ROS depending on the context, and that NRF2 and its redox targets are responsible for mediating ROS-dependent changes in stem cell status.

On a similar note, many of the critical enzymes involved in glutathione synthesis, as well as phase, I, II, and III xenobiotic/drug metabolism are regulated by NRF2 (Dodson et al., 2019). Accordingly, high levels of glutathione have been shown to be required for MSC function, and increased expression of the catalytic and modulatory subunits of glutamate cysteine ligase (GCLC/GCLM), glutathione reductase (GR), and thioredoxin-1 (TXN1) have all been associated with the increased ability of MSCs to self-renew, migrate, and mitigate the effects of pro-inflammatory stimuli/oxidative insult, particularly when transplanted as a form of immunotherapy (Jeong et al., 2018; Zhang et al., 2018; Lim et al., 2020). High glutathione and glutathione peroxidase 2 (GPX2) levels have also been reported in iPSCs, with depletion of GPX2 resulting in increased ROS-dependent DNA damage (Dannenmann et al., 2015). Knockdown of microsomal glutathione-S-transferase (MGST1) inhibits the differentiation of mouse HSCs but increases their proliferative/stem-like state (Bräutigam et al., 2018). Even the NRF2 target ATP binding cassette subfamily G member 2 (ABCG2), which may be involved in the export of glutathione conjugated substrates, is highly expressed in primitive HSCs, but downregulated upon differentiation, with forced overexpression in HSPCs preventing differentiation and resulting in HSPC depletion in the bone marrow and peripheral blood (Zhou et al., 2001; Kim et al., 2002). Thus, ABCG2 may play a role not only in mediating HSC/HSPC self-renewal and differentiation, but also in promoting their survival against cytotoxic insult. Overall, these studies indicate that NRF2-dependent regulation of redox homeostasis plays a critical role in mediating the ability of different stem cell types to differentiate or survive oxidative insult, and that while little is known in the context of NSCs and aging, they may rely on similar pathways that gradually decline with age.

#### Stem Cell Metabolism Influenced by NRF2

Along with its regulation of redox homeostasis, NRF2 also controls the expression of genes involved in carbohydrate, lipid, and mitochondrial metabolism. An example of this discussed earlier is the NRF2-dependent upregulation of HIF-1α in NSCs, which redirects glucose away from glycolysis into the PPP (Hawkins et al., 2016). While the connection between NRF2 and HIF-1α activation is well-established, direct transcriptional activation of HIF-1α has remained somewhat controversial. However, a recent study indicated that HIF-1α does have a functional ARE in its promoter, inferring that at least in certain contexts HIF-1α could be a direct target gene of NRF2 (Lacher et al., 2018). In terms of the role of the PPP in stem cell function, a number of studies have shown that loss of glucose-6-phosphate dehydrogenase (G6PD) increases mouse ESC susceptibility to oxidative stress-induced apoptosis, as well as effects their ability to undergo erythropoietic differentiation (Fico et al., 2004; Paglialunga et al., 2004). In addition to ESCs, cardiac progenitor cells (CPCs) from diabetic patients have been shown to exhibit decrease enzymatic activity of not only G6PD, but also transketolase (TKT) another NRF2 target gene (Agyeman et al., 2012), resulting in ROS production and apoptosis (Katare et al., 2013). As G6PD deficiencies have been reported in not only type I and II diabetes, but also aging and neurodegenerative diseases, loss of this key PPP enzyme could be a critical driver of stem cell depletion during injury or disease.

NRF2 has also been shown to play an important role in overseeing mitochondrial biogenesis and metabolism, although direct confirmation of target genes involved in these processes has been lacking (Dinkova-Kostova and Abramov, 2015). NRF2 has been shown to activate mitochondrial biogenesis through transcriptional upregulation of nuclear respiratory factor 1 (NRF1), which activates transcription factor A, mitochondrial (TFAM) to initiate the production of new mitochondria (Piantadosi et al., 2008). Additionally, the promoter of peroxisome proliferator-activated receptor gamma, coactivator 1 alpha (PGC-1α/PPARG) has two AREs, although they have not been validated as functional, and has been reported to be both up- and downregulated by NRF2 activation depending on the context (Clark and Simon, 2009; Dinkova-Kostova and Abramov, 2015). In the case of stem cell maintenance, the expression levels of NRF1, PGC-1α, and TFAM all increase during differentiation of MSCs (Chen et al., 2008). PGC-1α expression and increased mitochondrial biogenesis have also been observed in NSCs differentiated into motor neurons (O’Brien et al., 2015). Furthermore, both NRF1 and PGC-1α are upregulated in NSCs transplanted into the hippocampus of amyloid precursor protein /presenilin Alzheimer’s transgenic mice, which increased the number of mitochondria, and thus could serve as a means to preserve hippocampal function during AD progression (Zhang W. et al., 2015).

With regards to NRF2-dependent regulation of mitochondrial metabolism, the mRNA levels of carnitine palmitoyl transferase 1A and 2A (CPT1/2), which catalyze the rate limiting step of fatty acid oxidation, are decreased in NRF2 knockout cell lines and mice (Meakin et al., 2014; Pang et al., 2014). NRF2 has also been shown to directly regulate the expression of numerous mitochondrial respiratory chain subunits, including ATP synthase subunit alpha, cytochrome c oxidase subunit NDUFA4, as well as cytochrome oxidase subunits 2 and 4l1 (COX2/COX4l1) (Agyeman et al., 2012; Holmström et al., 2013). This correlates with the high NRF2 levels and increased fatty acid oxidation observed in quiescent versus activated adult stem cells and indicates that NRF2-dictated transcriptional events are critical drivers of increased reliance on mitochondrial function. Intriguingly, NRF2 has been shown to suppress numerous aspects of liver lipid metabolism, as the levels of a variety of lipogenic and lipid catabolizing enzymes were suppressed in Keap1 knockdown, hepatocyte Keap1 null, and CDDO-Im treated mice (Wu et al., 2011). Opposite to NRF2-dependent upregulation of fatty acid oxidation during quiescence, this fits NRF2 downregulation being associated with increased lipogenesis during NSC differentiation. Thus, NRF2 activation of mitochondrial metabolism and suppression of lipid-related target genes clearly plays a role in stem cell maturation across multiple organ systems.

#### NRF2 Regulation of Stem Cell Epigenetics, Transcription, and Inflammation

Discussed in detail above, both SIRT1 and SIRT6 have been shown to regulate transcription and stemness in an NRF2-dependent manner in a number of different stem cell types, including NSCs. This infers that NRF2 can regulate stem cell self-renewal and initiation of differentiation programs via control of epigenetic factors and other transcriptional regulators. This is further supported by the fact that NRF2 suppression of *OCT4* and *NANOG*, two transcription factors that play a critical role in dictating stem cell pluripotency, is required for hESCs to differentiate towards a neuroectodermal fate. Interestingly, activation of NF-κB and a pro-inflammatory response has been shown to inhibit MSC and HSC differentiation and function (Chang et al., 2013; Ping et al., 2019; Ramalingam et al., 2020). Conversely, NF-κB activation is required for NSC differentiation, and inhibition of this pathway keeps NSCs in an undifferentiated state (Zhang Y. et al., 2012). It has long been established that NRF2 and NF-κB generally have an inverse relationship (Wardyn et al., 2015). Thus, in NSCs, downregulation of NRF2 and upregulation of the NF-κB cascade promote differentiation, with the inverse promoting NSC self-renewal. This relationship may not hold true in other adult stem cell types, such as MSCs and HSCs where high NF-κB levels prevent differentiation; however, it is clear that NRF2 control of other transcriptional responses appears to play an integral role in dictating stem cell stemness, proliferation, and differentiation, particularly in the context of NSC-driven neurogenesis.

## Conclusion and Perspectives

The ability of stem cells to maintain a constant balance between quiescence, self-renewal, proliferation, and differentiation is key to preventing stem cell exhaustion and aging. Although redox and metabolic processes are known to be at the core of stem cell activity and function, the governing molecular mechanisms have not been well defined. There is increasing evidence for a central role of the NRF2-transcription factor in controlling redox and metabolic pathways in stem cells. Evidence in several model systems, and across different tissue types, suggest that NRF2 is fundamentally involved in the delicate endeavor of maintaining lifelong stem cell homeostasis. From this perspective, NRF2’s classical role in regulating the stress response can now be revised to include the basic function of stem cells. Delineating the precise nature and scope of such nascent but vital NRF2-based processes will not only reveal fundamental aspects of stem cell biology, but also support the development of innovative strategies to advantageously modulate stem cell function during ageing and disease.

From a basic science point of view, since NRF2 plays unique lineage- and subtype-specific roles in regulating stem cell activity, fate and function, it will be essential to investigate the precise mechanisms underlying these NRF2 processes in the future. It will also be important to understand how lifespan changes in the stem cell environment may impact NRF2’s intrinsic control of stem cell function. From a clinical lens, determining the optimal ways to modulate the NRF2 pathway in stem cells, *ex vivo* for cell transplantation, or in directly *in vivo* through the development of effective small molecule inhibitors acting upstream or downstream of NRF2, will be critical. Such approaches, as tailored to specific stem cell types, age and disease context will likely expand opportunities for clinical translation. Given that stem cells are core drivers of organismal aging, health and longevity, a greater understanding of these NRF2-based aspects may make it possible one day to rejuvenate tissues in older individuals, thereby increasing human healthspan and perhaps lifespan.
